# Valgus Stress Radiography as a Criterion for Total Ankle Arthroplasty Without Medial Malleolar Osteotomy: A Report of Three Severe Varus Cases

**DOI:** 10.7759/cureus.98222

**Published:** 2025-12-01

**Authors:** Yuki Etani, Takaaki Noguchi, Seiji Okada, Ken Nakata, Kosuke Ebina

**Affiliations:** 1 Department of Sports Medical Biomechanics, The University of Osaka Graduate School of Medicine Faculty of Medicine, Suita, JPN; 2 Department of Orthopaedic Surgery, The University of Osaka Graduate School of Medicine Faculty of Medicine, Suita, JPN; 3 Department of Health and Sport Sciences, The University of Osaka Graduate School of Medicine Faculty of Medicine, Suita, JPN

**Keywords:** ankle, osteoarthritis, rheumatoid arthritis, total ankle arthroplasty, varus

## Abstract

Total ankle arthroplasty (TAA) is performed for ankle arthritis; however, in cases with severe varus deformity, malalignment of implant positioning, and early loosening can be problematic. Medial malleolar osteotomy is often performed in such cases to facilitate proper implant positioning, but it carries risks including nonunion and delayed rehabilitation. This study reports three cases of TAA for severe varus ankle deformity (preoperative talar tilt >15°) performed without medial malleolar osteotomy, using preoperative valgus stress radiography as the selection criterion. Based on our clinical experience, we hypothesized that in cases where varus deformity can be corrected (talar tilt ≤10°) under valgus stress (a provisional threshold defined empirically), acceptable short-term outcomes might be achieved without osteotomy. Accordingly, three elderly women (aged 73-78 years; one with rheumatoid arthritis, two with osteoarthritis) meeting this criterion underwent TAA and were followed for one year. Standard rehabilitation was initiated in all but one case, requiring a tailored rehabilitation program due to concomitant surgery. Minor complications included two suspected or nondisplaced medial malleolar fractures, both healing without medical intervention or unloading. At final follow-up, all patients achieved pain relief, improved mobility, and satisfactory radiographic alignment without implant loosening. Preoperative valgus stress radiography may assist in identifying selected patients who can undergo TAA without medial malleolar osteotomy. However, the high incidence of medial malleolar fracture in this small cohort indicates that the provisional 10° cutoff should be interpreted with caution and may not be sufficiently conservative. Larger studies are needed to determine a safer and more appropriate threshold for omitting medial malleolar osteotomy.

## Introduction

Total ankle arthroplasty (TAA) is an established surgical option for end-stage ankle osteoarthritis (OA) and rheumatoid arthritis (RA), and is considered particularly suitable for elderly patients or those with low functional demand [1,2]. However, its applicability in patients with severe varus deformity (talar tilt >15°) has been questioned, as such cases are associated with higher complication and revision rates [3,4]. To address this issue, medial malleolar osteotomy in conjunction with TAA has been advocated to correct coronal malalignment and facilitate proper implant positioning [5]. Although effective in certain cases, this procedure carries risks such as displacement or nonunion at the osteotomy site and implant failure [6].

Avoiding medial malleolar osteotomy when it is not strictly necessary may lead to better outcomes by reducing surgical morbidity and enabling earlier rehabilitation. Nevertheless, clear criteria to determine when medial malleolar osteotomy is required, and when it can be safely omitted, have not been firmly established.

We hypothesized that, in patients whose talar tilt could be corrected to ≤10° under valgus stress, favorable outcomes could be achieved without the procedure. This threshold was provisionally defined based on our clinical experience and should be validated in future studies. In this report, we present three cases of severe varus ankle deformity in which valgus stress radiography demonstrated adequate correction of the talar tilt to ≤10°. All patients underwent TAA without concomitant medial malleolar osteotomy and achieved favorable short-term clinical and radiographic outcomes.

This research was conducted in accordance with the tenets of the Declaration of Helsinki and was approved by the Institutional Ethics Review Board of Osaka University Hospital (approval number: 14219). Written informed consent for publication of this case report and any accompanying images was obtained from all patients.

## Case presentation

The patients’ demographics are shown in Table 1. All three patients were elderly women (73-78 years) with either OA or RA of the ankle, presenting with severe varus deformity. Preoperative mobility was markedly limited, with walking ability ranging from five to 10 minutes using an assistive device. Preoperative weight-bearing radiographs demonstrated talar tilts of 16°-20° varus, each improving to ≤10° varus under valgus stress. Valgus stress radiography was performed manually. With the lower leg stabilized, the examiner applied maximal manual valgus force to the hindfoot during image acquisition.

**Table 1:** Clinical characteristics and radiographical outcomes/findings of three cases. OA: osteoarthritis, RA: rheumatoid arthritis, DAS: Disease Activity Score, TAA: total ankle arthroplasty, LCL: lateral column lengthening, SAFE-Q: self-administered foot evaluation questionnaire.

Variable	Case 1	Case 2	Case 3
Age (year)	77	78	73
Sex	F	F	F
Preoperative diagnosis	OA	RA	OA
DAS28-CRP	-	3.24	-
Duration of Follow up (month)	12	12	12
Body Mass Index	20.4	26.9	25.2
Preoperative dorsiflexion of ankle (degrees)	5	15	10
Preoperative plantarflexion of ankle (degrees)	30	30	20
Postoperative dorsiflexion of ankle (degrees)	10	15	15
Postoperative plantarflexion of ankle (degrees)	40	40	30
Preoperative talar tilt (degrees)	20 (varus)	20 (varus)	16 (varus)
talar tilt under valgus stress (degrees)	10 (varus)	8 (varus)	7 (varus)
Surgical technique	TAA+LCL+gastro-recession	TAA	TAA+Hallux valgus surgery
malleolar fracture under surgery	No	No	No
Postoperative malleolar fracture	No	Yes	Yes
Positioning of the tibial component at TAA (degrees)	2 (valgus)	1 (valgus)	1 (varus)
Prosthetic migration/loosening	No	No	No
Preoperative SAFE-Q score			
Pain	75.6	17.8	22.2
Activities of daily living	90.9	18.2	25
Social activity	91.7	12.5	12.5
General health	85	10	45
Shoe-related	75	41.7	41.7
Postoperative SAFE-Q score			
Pain	82.2	97.2	73.9
Activities of daily living	86.4	65.9	86.4
Social activity	100	83.3	87.5
General health	95	90	95
Shoe-related	83.3	83.3	91.7

All surgical procedures were performed using a mobile-bearing ankle prosthesis (FINE Total Ankle System; NAKASHIMA HEALTHFORCE Co.) via a modified anterolateral approach [7]. A tibial osteotomy was made perpendicular to the tibial axis approximately 10-11 mm proximal to the tibial plafond, and the talar osteotomy was performed according to the planned implant size to restore a normal tibio-calcaneal angle. Care was taken to avoid varus alignment of the tibial cut. To prevent postoperative medial shift of the weight-bearing axis, the tibial osteotomy was made perpendicular or in slight valgus relative to the tibial axis, and the tibial component was placed as laterally as possible within the safe range. The posterior talus was thoroughly released, and a gastrocnemius recession was performed if dorsiflexion was limited compared with the preoperative status [8]. To enhance the stability of the tibial component, a hydroxyapatite augmentation block was inserted into the tibial medullary canal before implant placement as described in our previous report [9]. In cases without concomitant procedures, range-of-motion (ROM) exercises were initiated on postoperative day 3, and full weight-bearing was initiated on day 7.

Case 1

**Figure 1:**
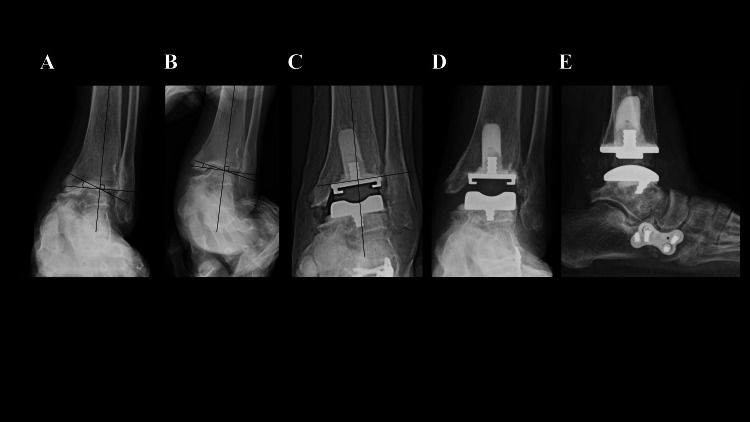
Ankle joint radiographs of Case 1. (A) Preoperative weight-bearing radiograph showing a 20° varus talar tilt. (B) Under valgus stress, the talar tilt is corrected to 10° varus. (C) Immediately after surgery, the tibial component was positioned in 2° of valgus. (D, E) At the final follow-up, weight-bearing anteroposterior and lateral radiographs of the ankle joint show no evidence of prosthetic loosening.

A 77-year-old woman with OA and progressive collapsing foot disease (PCFD) could walk for only 10 minutes with a T-cane because of ankle pain. Preoperative weight-bearing radiographs showed a talar tilt of 20° varus (Figure 1A), which was corrected to 10° varus under valgus stress (Figure 1B). She underwent TAA and a gastrocnemius recession, and a lateral column lengthening was additionally performed to alleviate symptoms related to PCFD; specifically, forefoot abduction and collapse of the medial longitudinal arch. The tibial component was placed in 2° valgus (Figure 1C). Rehabilitation was delayed due to concomitant procedures (ROM exercises at two weeks, half weight-bearing at three weeks, and full weight-bearing at four weeks). No notable postoperative complications occurred. At one year, she could walk for more than one hour without pain, with no evidence of prosthesis loosening (Figure 1D, 1E). Her total SAFE-Q (self-administered foot evaluation questionnaire) score improved from 418.2 to 446.9.

Case 2

**Figure 2:**
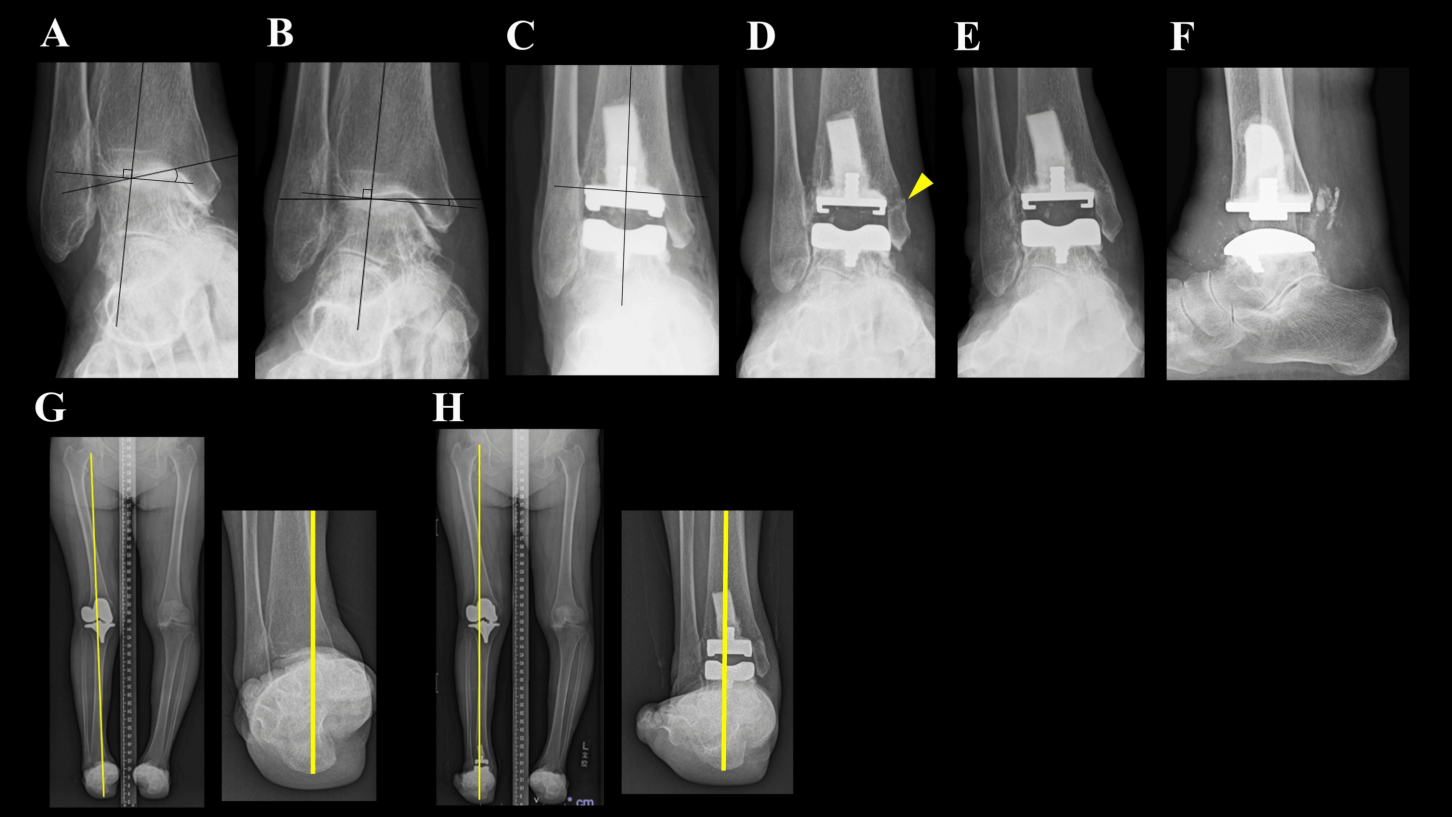
Ankle joint radiographs of Case 2. (A) Preoperative weight-bearing radiograph showing a 20° varus talar tilt. (B) Under valgus stress, the talar tilt is corrected to 8° varus. (C) Immediately after surgery, the tibial component was positioned in 1° of valgus. (D) Six weeks postoperatively, a suspected fracture was observed in the area indicated by the arrow. (E, F) At the final follow-up, weight-bearing anteroposterior and lateral radiographs of the ankle joint show no evidence of prosthetic loosening. (G) Preoperative lower limb load axis on the hindfoot coronal (HC) view, showing the axis passing through the medial side of the ankle joint. (H) Postoperative lower limb load axis on the HC view, showing the axis passing through the center of the ankle joint.

A 78-year-old woman with RA (disease duration: four years), classified as Steinbrocker stage IV and class II, was being treated with baricitinib 2 mg/day; however, her DAS28-CRP remained at 3.24, indicating moderate disease activity. She could walk for only five minutes with a T-cane. Preoperative weight-bearing radiographs demonstrated a talar tilt of 20° varus (Figure 2A), which was corrected to 8° varus under valgus stress (Figure 2B). She underwent TAA with the tibial component positioned in 1° valgus (Figure 2C). Standard rehabilitation was initiated (ROM at three days, full weight-bearing at seven days). At the first postoperative outpatient visit, six weeks after surgery, radiographs revealed changes suggestive of a medial malleolar fracture (Figure 2D). As the patient was completely asymptomatic, no therapeutic intervention was undertaken. At one year, she could walk for more than one hour without a cane, and the weight-bearing axis passed through the center of the ankle joint, and radiographs demonstrated complete healing of the nondisplaced medial malleolar fracture (Figures 2E-2H). Her total SAFE-Q score improved from 100.2 to 419.7.

Case 3

**Figure 3:**
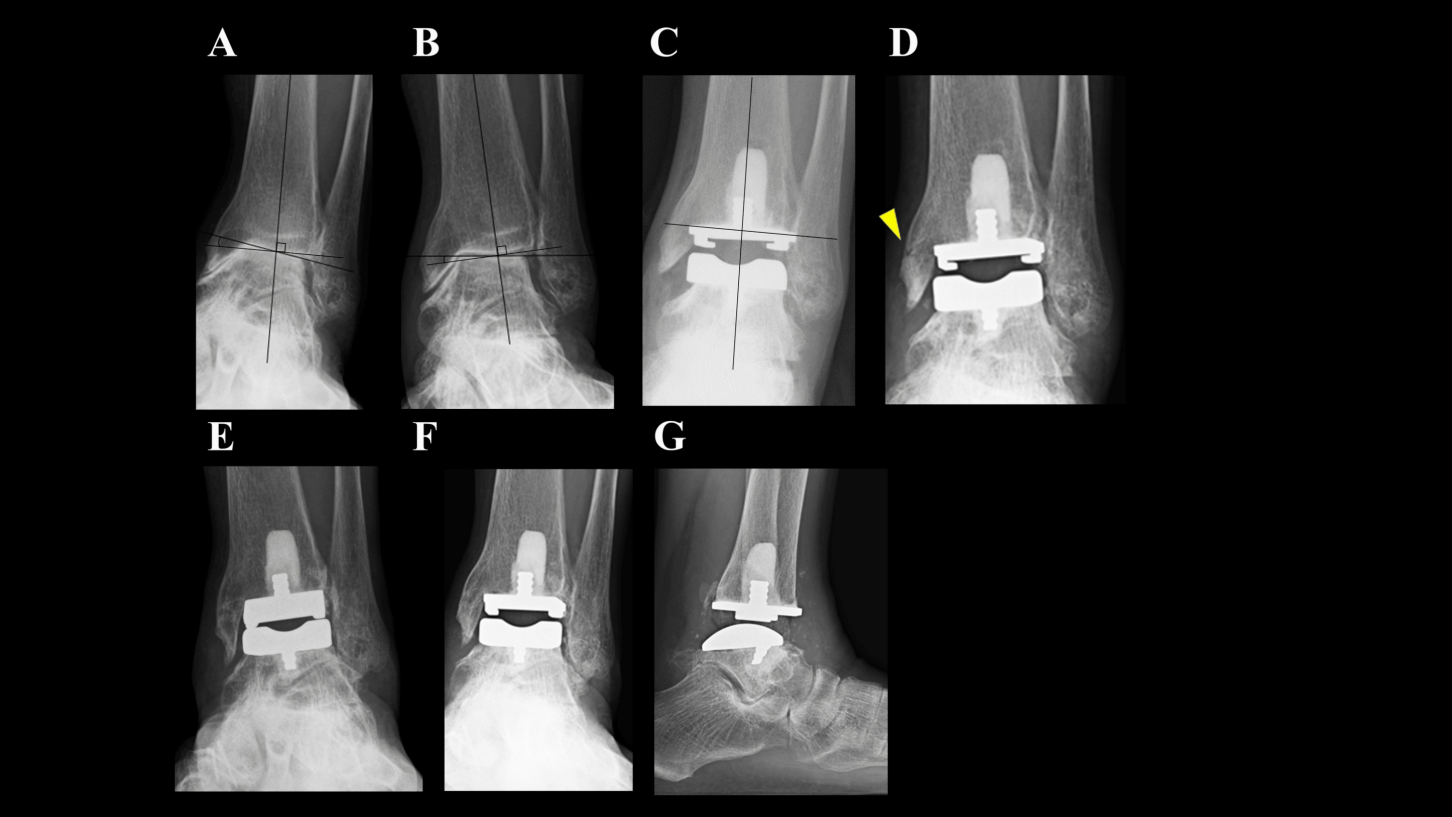
Ankle joint radiographs of Case 3. (A) Preoperative weight-bearing radiograph showing a 16° varus talar tilt. (B) Under valgus stress, the talar tilt is corrected to 7° varus. (C) Immediately after surgery, the tibial component was positioned in 1° of varus. (D) Two weeks postoperatively, a fracture was evident in the area indicated by the arrow. (E) At two months postoperatively, the fracture had completely healed. (F, G) At the final follow-up, weight-bearing anteroposterior and lateral radiographs of the ankle joint show no evidence of prosthetic loosening.

A 73-year-old woman with OA and a history of bipolar hip arthroplasty could walk for only 10 minutes with a handcart. Preoperative weight-bearing radiographs showed a talar tilt of 16° varus (Figure 3A), which was corrected to 7° varus under valgus stress (Figure 3B). She underwent TAA combined with hallux valgus correction, with the tibial component positioned in 1° varus (Figure 3C). Although the osteotomy resulted in slight varus alignment, it was deemed acceptable due to satisfactory coronal balance at the time of implant placement. Standard rehabilitation was initiated (ROM at three days, full weight-bearing at seven days). Two weeks postoperatively, a nondisplaced fracture of the medial malleolus was identified (Figure 3D). Given the minimal pain, gait training was continued without specific medical intervention. The fracture achieved union without displacement within two months (Figure 3E). At one year, she could walk for more than 30 minutes without a cane, with no evidence of prosthesis loosening (Figures 3F, 3G). Her total SAFE-Q score improved from 146.4 to 434.5.

## Discussion

Medial malleolar osteotomy in conjunction with TAA has been reported as an effective surgical option for ankles with severe varus deformity [5]. However, this approach is associated with potential complications such as nonunion at the osteotomy site and prosthetic loosening [6]. Furthermore, because postoperative weight-bearing is typically delayed, there is a risk of prolonged hospitalization and deterioration of physical function, which is of particular concern in elderly patients, the group most often indicated for TAA. We have previously reported that, in cases without medial malleolar osteotomy, full weight-bearing ambulation can be initiated as early as one week postoperatively [10]. Therefore, avoiding medial malleolar osteotomy in cases where it is unnecessary may be advantageous. Until now, however, there has been no established criterion for determining when this additional procedure can be safely omitted. Although the present study is limited to three cases with short-term follow-up, our findings suggest that in ankles where preoperative valgus stress radiography demonstrates correction of the talar tilt angle to ≤10°, TAA without medial malleolar osteotomy can yield favorable early outcomes. Establishing such a preoperative assessment criterion could be of substantial clinical value in guiding surgical decision-making.

In this series, the decision to omit medial malleolar osteotomy was based on whether varus deformity could be corrected on preoperative valgus stress radiographs. In cases where correction is not achievable, excessive tension in the medial supporting structures, particularly the deltoid ligament, may predispose to malpositioning of the implant in varus or excessive stress on the medial malleolus, potentially resulting in postoperative fracture [11]. In such cases, medial malleolar osteotomy is considered useful to achieve proper alignment and avoid excessive loading of the medial column. Conversely, in cases where valgus stress radiography demonstrates correction of the talar tilt angle to within 10°, as in our patients, the deltoid ligament is not excessively tense, allowing for satisfactory implant positioning without the need for osteotomy. In situations with marked ligamentous laxity, careful adjustment of the tibial resection depth and polyethylene insert thickness is essential to achieve appropriate coronal balance and ensure stability [12]. In certain cases, ankle arthrodesis may be considered as an alternative.

Among the three cases in this study, postoperative medial malleolar fractures occurred in two. This is an important finding and should not be regarded as a trivial or acceptable complication. Nevertheless, both fractures were nondisplaced, minimally symptomatic, and healed uneventfully without any modification of the rehabilitation protocol or need for additional intervention. In this sense, these fractures may still be less severe than the well-recognized complications associated with intentional medial malleolar osteotomy, such as nonunion, displacement, or delayed postoperative weight-bearing. However, the occurrence of these fractures suggests that our current threshold of ≤10° correction on valgus stress radiography may not yet represent a sufficiently safe margin. This finding indicates that a stricter cutoff (for example, requiring correction to ≤5°) might be more appropriate, and further investigation with a larger number of cases is warranted.

A key technical point in severe varus deformity is to avoid varus alignment of the distal tibial resection. Varus placement of the tibial component shifts the load axis medially, increasing contact stress on the medial prosthetic interface and the risk of early loosening. In our series, the tibial cut was planned to be made perpendicular (or slightly valgus) to the tibial axis, and the tibial component was placed as laterally as safely possible to maintain or lateralize the weight-bearing axis. However, precise coronal alignment cannot be measured intraoperatively, as no device allows exact quantification of varus-valgus angle during the tibial cut. In Case 3, the cut was judged neutral using an extramedullary alignment rod, but postoperative radiographs demonstrated 1° of varus.

Preservation of subtalar joint motion can help maintain appropriate coronal alignment and load distribution after TAA. In all three cases in this study, imaging confirmed that the subtalar joint was not ankylosed, which may have contributed to the favorable postoperative alignment observed. Further studies with a larger sample size are warranted to assess the relationship between subtalar joint mobility and long-term prosthesis stability in severe varus deformity.

Finally, the surgeon's experience is a critical factor for success in severe varus deformities [13], and such cases should be managed by highly proficient orthopedic surgeons.

This study has several limitations. First, it included only three cases with a one-year follow-up, which limits the generalizability of the findings and might be insufficient to adequately assess long-term implant survival. Second, the selection criterion was based solely on preoperative valgus stress radiography, without assessment of its measurement accuracy or interobserver reliability. Moreover, all patients in this series had a talar tilt angle ≤10° under valgus stress; therefore, outcomes in patients exceeding this threshold remain unknown. Given these limitations, our study should be regarded as a preliminary, hypothesis-generating observation. Future multi-center studies with larger cohorts and longer follow-up are warranted to validate the proposed criterion and clarify its clinical applicability.

## Conclusions

In the three cases where preoperative valgus stress radiography demonstrated correction of the talar tilt to ≤10°, TAA without medial malleolar osteotomy achieved stable short-term outcomes without implant-related complications, suggesting that this assessment method may be useful for determining when osteotomy can be avoided. However, nondisplaced medial malleolar fractures occurred in two of the three cases, indicating that the currently proposed 10° cutoff may not be sufficiently conservative. Larger studies are required to establish a more appropriate and safe threshold for selecting candidates for TAA without osteotomy.

## References

[1] Haskell A, Mann RA (2004). Ankle arthroplasty with preoperative coronal plane deformity: short-term results. Clin Orthop Relat Res.

[2] Shock RP, Christensen JC, Schuberth JM (2011). Total ankle replacement in the varus ankle. J Foot Ankle Surg.

[3] Doets HC, Brand R, Nelissen RG (2006). Total ankle arthroplasty in inflammatory joint disease with use of two mobile-bearing designs. J Bone Joint Surg Am.

[4] Usuelli FG, Di Silvestri CA, D'Ambrosi R, Orenti A, Randelli F (2019). Total ankle replacement: is pre-operative varus deformity a predictor of poor survival rate and clinical and radiological outcomes?. Int Orthop.

[5] Doets HC, van der Plaat LW, Klein JP (2008). Medial malleolar osteotomy for the correction of varus deformity during total ankle arthroplasty: results in 15 ankles. Foot Ankle Int.

[6] van der Plaat LW, Doets HC, van Dijk CN, Haverkamp D (2022). Medial malleolar osteotomy for the correction of tibiotalar varus deformity during total ankle arthroplasty: results in 95 ankles. Foot (Edinb).

[7] Hirao M, Ebina K, Etani Y (2021). Modified anterolateral approach for total ankle arthroplasty. Foot Ankle Orthop.

[8] Cychosz CC, Phisitkul P, Belatti DA, Glazebrook MA, DiGiovanni CW (2015). Gastrocnemius recession for foot and ankle conditions in adults: Evidence-based recommendations. Foot Ankle Surg.

[9] Shi K, Hayashida K, Hashimoto J, Sugamoto K, Kawai H, Yoshikawa H (2006). Hydroxyapatite augmentation for bone atrophy in total ankle replacement in rheumatoid arthritis. J Foot Ankle Surg.

[10] Sakata M, Hirao M, Noguchi T (2024). Early full weight-bearing and gait exercise after cemented total ankle arthroplasty with a modified anterolateral approach. Mod Rheumatol.

[11] Okamura G, Hirao M, Noguchi T (2024). Nonunion after medial malleolar osteotomy in total ankle arthroplasties for severe varus deformity: a report of 3 cases. J Surg Case Rep.

[12] Noguchi T, Hirao M, Okamura G (2024). Stabilizing effect of total ankle arthroplasty by distal translation and lateralization of talus in varus ankle deformity. Musculoskelet Surg.

[13] Usuelli FG, Maccario C, Pantalone A, Serra N, Tan EW (2017). Identifying the learning curve for total ankle replacement using a mobile bearing prosthesis. Foot Ankle Surg.

